# Outcomes of Inpatient Versus Outpatient Elective Foot and Ankle Surgery

**DOI:** 10.7759/cureus.4058

**Published:** 2019-02-12

**Authors:** Samuel R Huntley, Andrew S McGee, John L Johnson, Henry A Debell, Haley M McKissack, Gerald McGwin, Sameer M Naranje, Ashish Shah

**Affiliations:** 1 Miscellaneous, Miller School of Medicine, University of Miami, Miami, USA; 2 Orthopaedics, University of Alabama School of Medicine, Birmingham, USA; 3 Miscellaneous, University of Alabama School of Medicine, Birmingham, USA; 4 Epidemiology, University of Alabama School of Medicine, Birmingham, USA

**Keywords:** foot surgery, ankle surgery, outcomes, elective surgery, outpatient surgery, inpatient surgery, surgical outcomes

## Abstract

Background

Complications following orthopedic surgeries are undesirable and costly. A potential method to reduce these costs is to perform traditionally inpatient surgical procedures in the outpatient setting. The purpose of this study is to compare outcomes between inpatient and outpatient settings for elective foot and ankle surgeries using the National Surgical Quality Improvement Program (NSQIP) database.

Methods

Patients with Current Procedural Terminology (CPT) codes specific to orthopedic foot and ankle surgery were identified from the 2011-2015 American College of Surgeons NSQIP database. Demographics, comorbidities, and complications were compared between patients undergoing inpatient and outpatient procedures.

Results

Patients receiving inpatient surgery were significantly older and more frequently male. Black patients were significantly more likely to undergo inpatient surgery than outpatient surgery while white patients were significantly more likely to undergo outpatient surgery. Outpatients had a significantly higher mean body mass index (BMI) than inpatients. Smokers were at a significantly greater risk of undergoing inpatient surgery than outpatient surgery. Outpatients had significantly longer operative times, were more likely to receive general anesthesia, had a lower American Society of Anesthesiologists (ASA) class, were more likely to be functionally independent, and were less likely to expire postoperatively. Patients who received surgery as an inpatient were significantly more likely to have comorbidities as compared to outpatients. The overall risk of surgical complications was significant between groups with 8.6% in the inpatient group and 2.0% in the outpatient group. The overall risk of medical complications was 16.9% in the inpatient group and 1.7% in the outpatient group. Similar to the surgical complications, inpatients were significantly more likely to sustain each of the individual medical complications except for stroke/CVA and venous thromboembolism.

Conclusions

Outpatient management is associated with decreased postoperative complications in select patients. Performing more operations in the outpatient setting in select patients may be beneficial for cost reduction and patient satisfaction.

## Introduction

Medical and surgical complications following orthopedic surgeries, such as surgical site infection (SSI), venous thromboembolism (VTE), pneumonia (PNA), urinary tract infections (UTI), hospital readmission, and reoperation are undesirable, costly, and often grounds for imposing financial penalties on hospitals. For example, one study using the Veterans Affairs Surgical Quality Improvement Program database found patients with SSI following orthopedic surgeries had one of the highest mean costs attributable to SSI, with a risk-adjusted cost difference from those without SSI of $15,243 [1].Of the orthopedic subspecialties, the rate of SSI in foot and ankle surgeries is higher than other orthopedic procedures, given the nature of the microbial flora of the foot and the higher prevalence of foot and ankle abnormalities in patients with diabetes mellitus [2-4]. Numerous efforts have been made to reduce SSI and other complications associated with foot and ankle surgeries in order to improve outcomes and reduce spending. Along with this increased emphasis on minimizing the costs of healthcare, performing traditionally inpatient surgical procedures in the outpatient setting is a valuable alternative if found to be as safe as inpatient surgeries.

Several studies have demonstrated the safety and reduced costs of common orthopedic surgeries, including primary shoulder arthroplasty, hip arthroplasty, and knee arthroplasty, performed in the outpatient setting in selected patients [5-7]. Furthermore, there is evidence supporting the safety of outpatient total ankle arthroplasty and surgery for closed ankle fractures [8-10].Although the literature shows promising trends of safety in areas of outpatient orthopedic surgeries, it lacks any large database studies evaluating common elective foot and ankle surgeries.

Given the current increasing costs of health spending, reimbursements tied to hospital performance, the astounding cost of complications that occur at greater rates in foot and ankle subspecialties, the increasing number of foot and ankle procedures performed yearly, and the lack of literature, further investigation into the safety of outpatient foot and ankle surgeries is an important area of interest. The purpose of this study was to evaluate surgical outcomes between the inpatient and outpatient settings for elective foot and ankle surgeries using the National Surgical Quality Improvement Program (NSQIP) database.

## Materials and methods

Data source

The American College of Surgeons National Surgical Quality Improvement Program (NSQIP) database is commonly used in the orthopedic surgery literature. In the NSQIP database, trained clinical reviewers prospectively collect preoperative, intraoperative, and postoperative data through a standardized methodology. The NSQIP database was queried for any patient who received at least one of 218 unique CPT codes specific to orthopedic foot and ankle surgery. A total of 27 Current Procedural Terminology (CPT) codes were returned, representing our sample of 7,672 patients. Any codes associated with foot or ankle fractures (27814, 27792, 27822, 27766, 27823, 27769, 28445) were excluded, as these are not elective procedures.

Variables

Demographic information included age, sex, body mass index (BMI), race, and smoking status. Hospital and operative variables included: mean operative time, use of general anesthesia, total hospital length of stay, American Society of Anesthesiologists (ASA) classification, functional status, and discharge destination. Ten different comorbidities were examined, including acute renal failure, ascites, bleeding disorders, congestive heart failure (CHF), chronic obstructive pulmonary disease (COPD), diabetes mellitus (DM), dialysis, dyspnea, hypertension (HTN), and steroid use. The complications of interest included surgical complications (i.e., superficial wound infection, deep incisional surgical site infection, and wound disruption) and medical complications (i.e., pneumonia, reintubation, failure to wean from ventilator, progressive renal insufficiency, acute renal failure, urinary tract infection, myocardial infarction, cardiac arrest, stroke, blood transfusion, venous thromboembolism, sepsis, septic shock, and reoperation).

Statistical analysis

The primary outcome of interest for this analysis was the risk of surgical and medical complications. T- and chi-square tests were used to compare demographic characteristics, comorbidities, and complications between patients undergoing inpatient and outpatient procedures. Logistic regression was used to adjust the association between the surgery groups and surgical and medical complications for specific demographic and surgical characteristics and comorbidities. P-values of <0.05 were considered statistically significant. This research did not receive any specific grant from funding agencies in the public, commercial, or not-for-profit sectors.

## Results

The demographic and hospital variables of our sample are shown in Table 1. Patients receiving inpatient surgery were significantly older and more frequently male. Black patients were significantly more likely to undergo inpatient surgery than outpatient surgery while white patients were significantly more likely to undergo outpatient surgery. Outpatients had a significantly higher mean BMI than inpatients. Smokers were at a significantly greater risk of undergoing inpatient surgery than outpatient surgery. With regard to operative variables, outpatients had significantly longer operative times, were more likely to receive general anesthesia, had a lower ASA class, were more likely to be functionally independent, and were less likely to expire postoperatively.

**Table 1:** Demographics and hospital variables of 7,672 patients undergoing orthopedic foot and ankle surgery stratified by inpatient status BMI: body mass index; ASA: American Society of Anesthesiologists

Variable	Inpatient	Outpatient	p-value
Female Gender (%)	33.9	43.0	<0.001
Mean Age (± S.D.)	61.8 ± 13.9	47.8 ± 16.7	<0.001
Race (%)			
Asian	1.6	1.4	<0.001
Black	22.0	9.2	
Hispanic	8.1	7.2	
Native American	1.1	0.9	
Other/Unknown	10.8	15.2	
White	56.5	66.2	
Mean BMI (± S.D.)	29.5 ± 7.5	30.7 ± 7.3	<0.001
Current Smoker (%)	23.4	18.1	<0.001
Mean Operative Time, Minutes (± S.D.)	66.1 ± 56.1	73.9 ± 50.2	<0.001
General Anesthesia (%)	70.7	83.4	<0.001
Elective Surgery (%)	44.7	95.4	<0.001
ASA Class (%)			
1,2	16.8	69.2	<0.001
3,4,5	83.2	30.8	
Functional Status (%)			
Independent	77.5	96.6	<0.001
Partially/Totally Dependent	22.5	3.4	
Discharge Destination (%)			
Expired	1.6	0.0	<0.001
Home	64.1	97.7	
Not Home	34.3	2.3	

The comorbidity variables in our sample are shown in Table 2. Patients who received surgery as an inpatient were significantly more likely to have each of the 10 comorbidities as compared to an outpatient.

**Table 2:** Comorbidities of 7,672 patients undergoing orthopedic foot and ankle surgery stratified by inpatient status COPD: Chronic obstructive pulmonary disease

Variable	Inpatient %	Outpatient %	p-value
Acute Renal Failure	3.8	0.3	<0.001
Ascites	0.6	0.0	<0.001
Bleeding Disorder	19.7	5.2	<0.001
Congestive Heart Failure	5.4	0.3	<0.001
COPD	7.0	2.1	<0.001
Diabetes	63.4	18.6	<0.001
Dialysis	16.0	2.7	<0.001
Dyspnea	11.0	3.4	<0.001
Hypertension	73.4	34.7	<0.001
Steroid Use	6.5	2.4	<0.001

The overall risk of surgical complications (i.e., superficial, deep surgical site infections and wound disruptions) was 8.6% in the inpatient group and 2.0% in the outpatient group (p<0.001). Inpatients were significantly more likely to sustain pooled and individual surgical complications as compared to outpatients. The overall risk of medical complications was 16.9% in the inpatient group and 1.7% in the outpatient group. Similar to the surgical, inpatients were significantly more likely to sustain each of the individual medical complications (p<0.001) except for stroke/cerebrovascular accident (CVA) (p=0.138) and venous thromboembolism (p=0.394). Complications are shown in Table 3.

**Table 3:** Medical and surgical complications of 7,672 patients undergoing orthopedic foot and ankle surgery stratified by inpatient status CPR: cardiopulmonary resuscitation; CVA: cerebrovascular accident

Variable	Inpatient %	Outpatient %	p-value
Surgical complications	8.6	2.0	<0.001
Superficial wound infection	3.8	1.2	<0.001
Deep incisional surgical site infection	3.7	0.7	<0.001
Wound disruption	1.5	0.3	<0.001
Medical complications	16.9	1.7	<0.001
Pneumonia	2.1	0.1	<0.001
Reintubation	1.9	0.1	<0.001
Failure to wean from ventilator	1.5	0.0	<0.001
Progressive renal insufficiency	0.9	0.1	<0.001
Acute renal failure	0.9	0.0	<0.001
Urinary tract infections	1.7	0.4	<0.001
Myocardial infarction	0.9	0.2	<0.001
Cardiac arrest requiring CPR	1.1	0.1	<0.001
Stroke/CVA	0.2	0.1	0.138
Bleeding requiring transfusion	6.8	0.3	<0.001
Venous thromboembolism	0.5	0.4	0.394
Sepsis	5.0	0.6	<0.001
Septic shock	1.6	0.1	<0.001
Reoperation	17.8	2.2	<0.001

After adjusting for various confounders on a multivariable analysis in Table 4, inpatients had 3.72 times greater odds of suffering a medical complication (p<0.001). As shown in Table 5, inpatients had 1.81 times greater odds of suffering a surgical complication as compared to outpatients (p=0.006). All variables included in the models were significant predictors of complications.

**Table 4:** Predictors of medical complications on multivariable logistic regression ASA: American Society of Anesthesiologists

Variable	Adjusted Odds Ratio	95% CI	p-value
Inpatient (ref=outpatient)	3.72	2.42-5.73	<0.001
ASA class 3-5 (ref=1-2)	3.17	2.18-4.61	<0.001
Elective surgery (ref=no)	0.40	0.33-0.50	<0.001
Functionally Iindependent (ref=partially/totally dependent)	0.673	0.54-0.84	<0.001
Diabetic (ref=non-diabetic)	1.40	1.13-1.74	0.002
Bleeding disorder (ref=no)	1.49	1.20-1.85	<0.001
Ascites (ref=no)	2.74	1.07-6.99	0.035
Dialysis (ref=no)	1.74	1.39-2.19	<0.001

**Table 5:** Predictors of surgical complications on multivariable logistic regression ASA: American Society of Anesthesiologists

Variable	Adjusted Odds Ratio	95% CI	p-value
Inpatient (ref=outpatient)	1.81	1.19-2.75	0.006
Female Gender (ref=Male)	0.64	0.37-0.70	0.002
ASA Class 3-5 (ref=1-2)	3.68	2.22-6.10	<0.001
Diabetic (ref=non-diabetic)	1.96	1.43-2.70	<0.001

## Discussion

When evaluating outpatient versus inpatient surgery, it is important to recognize the benefits provided by each. Outpatient surgery reduces the cost and length of hospital stay and has higher patient satisfaction as compared to inpatient surgery. However, inpatient surgery offers more resources and more intensive postoperative care [11-12]. It is important to evaluate the safety of different operations in the outpatient setting in order to determine what patients may benefit from specific outpatient surgeries. This study evaluates risk factors and complication rates for inpatient versus outpatient elective ankle surgery.

A 2005-2013 ACS-NSQIP study performed by Qin and colleagues evaluated the safety and outcomes of 5,265 patients undergoing surgery for non-emergent, closed ankle fractures in the inpatient and outpatient setting. This study found that outpatients had significantly decreased chances of 30-day postoperative pneumonia and found no significant difference in surgical morbidity, reoperations, or readmissions [10]. These findings suggest that outpatient surgery for ankle fractures is acceptable in carefully selected patients. A study by Borenstein and colleagues evaluated complications in 65 patients receiving outpatient total ankle arthroplasty (TAA). This study’s results showed no emergency department visits or readmissions, and the overall complication rate reported by this study was 15.4%. These complications included one (1.5%) minor wound dehiscence, one (1.5%) wound infection, two (3%) revision surgeries, and eight (12%) non-revision surgeries. This study demonstrated that outpatient TAA is a safe and beneficial choice in appropriately selected patients [8].

Our results showed no significant difference (p=.394) between inpatient and outpatient populations for the risk of venous thromboembolism. Thromboembolism is rare in foot and ankle surgery, and the literature does not support the use of prophylaxis [10,13-14]. Outpatient rates of deep vein thrombosis (DVT) have been shown to be as low as 0.3%, and a randomized controlled trial by Lapidus et al. showed no difference in the rate of DVT and pulmonary embolism between a group that received prophylaxis and a group that did not [10,15]. However, a study by Shen et al. found an increased risk of DVT in inpatients as compared to outpatients [16]. Despite this difference from our results, the management does not change, as prophylaxis would still not be indicated [13-15]. Based on our results, we cannot conclude that inpatient ankle surgery is a risk factor for thromboembolic events. For this reason, thromboembolism should not preclude consideration for outpatient ankle surgery.

Based on our results, the most important factors for predicting inpatient vs outpatient management were ASA classification, race, and functional status. ASA had the largest disparity, with 52.4% more patients with ASA greater than or equal to 3 being managed as inpatients. ASA status is defined as follows: 1=“a normal healthy patient,” 2=“a patient with mild systemic disease,” 3=“a patient with severe systemic disease,” 4=“a patient with severe systemic disease that is a consistent threat to life,” and 5=“a moribund patient who is not expected to survive without the operation.” It has been shown to be a reliable predictor of complications and mortality following surgery [17]. In comparison to the outpatient group, inpatients had higher rates of diabetes (44.8%), hypertension (38.7%), and bleeding disorders (14.5%). These chronic systemic conditions directly contribute to higher ASA status as well as increased risk of medical and surgical complications, such as infection and reoperation. Nearly all (96.6%) patients with independent functional status were managed outpatient as compared to 77.5% of inpatients. Inherent to the surgical setting, the discharge destination had a predictable correlation, as more patients were discharged to home versus not home in the outpatient group. Additionally, more patients expired following surgery in the inpatient than in the outpatient group. These findings are consistent with a study by Visnjevac et al., which showed that functional status was an independent predictor of 30-day mortality within each ASA class [18]. Given our results, both ASA and functional status should be considered when determining inpatient vs outpatient management. Smoking was also shown to have a slightly increased (5.3%) inpatient burden, which may indicate that patients who smoke are more suited for inpatient operations. Interestingly, outpatients had a significantly higher mean BMI than inpatients. This implies that, to an extent, a higher BMI does not necessitate inpatient management. Twenty-two percent of inpatient surgeries were performed on black patients, as compared to only 9.2% of outpatient surgeries; black patients were 12.8% more likely to have inpatient management. White patients and patients of unknown race were 10% and 4.4% more likely to receive outpatient management, respectively. In summary, the demographic most likely to receive inpatient management would be an older black patient with an ASA classification greater than or equal to 3 and dependent functional status.

Our results showed that those undergoing inpatient surgery were significantly older than those undergoing outpatient surgery by an average of 14 years. Additionally, across all comorbidities studied, a significantly greater proportion of patients undergoing inpatient surgery had comorbidities as compared to outpatient surgery. Although causality cannot be concluded, it is likely that these comorbidities, as well as increased patient age, necessitate more extensive care, which can be provided in the inpatient setting. Complications were uniformly more common in the inpatient population as well. Surgical complications were 6.6% more likely while medical complications were 15.2% more likely. This correlates to the higher ASA status and more comorbidities seen in the inpatient population.

This study had several strengths worth mentioning. Due to the use of a large, multicenter database, we were able to have a substantially large and diverse patient population with 17,939 total patients. It represented over 400 hospitals with many more providers, suggesting a reasonable estimation of the demographics, comorbidities, and complication rates of the population undergoing foot and ankle surgery. To the authors’ knowledge, this is the first study of its size that broadly compares the complication rates of inpatient and outpatient foot and ankle surgery. Further validating our efforts, the NSQIP database is widely used and accepted by the medical research community as a tool to analyze and improve the quality of care provided both locally and nationally [19].

This study also had several limitations that should be noted. Although the collection of NSQIP data is held to a high standard [19], the authors did not personally collect the data, and there is no way of ensuring the accuracy of the records. Furthermore, a limitation inherent to the NSQIP database, any complications that may have occurred after the surgery were limited to 30 postoperative days, and, therefore, many complications could have occurred after this time frame and would lead to an underestimation of these rates. There was also no known specific criteria used to determine which patients would undergo surgery in each setting. This was likely determined by the clinical judgment of the physicians and the details determining those decisions were not included in the database. Some patients may have undergone multiple traumatic injuries that required inpatient stays. These patients may have received inpatient surgery because of this and, in other cases, they may have been candidates for outpatient surgery. We recognize the limitations of this study, but we believe that these results could be useful in developing further prospective studies and randomized controlled trials that compare the operative outcomes of inpatient and outpatient TAA.

## Conclusions

In conclusion, the results of this study suggest that outpatient management may reduce medical and surgical complication rates among certain patients undergoing elective foot and ankle surgery. ASA classification, race, and functional status may be important to consider in determining whether patients should receive inpatient or outpatient treatment. Due to the benefits of outpatient surgery, such as cost reduction and patient satisfaction, performing a higher proportion of these operations in the outpatient setting may be beneficial in select patients.
